# The Effectiveness and safety of T-MSAT on inpatients with acute low back pain caused by traffic accidents: A protocol for randomized controlled trial

**DOI:** 10.1097/MD.0000000000023851

**Published:** 2021-02-05

**Authors:** Jeong-Hun Han, Byung-Hak Park, Jin-Hun Park, Tae-Woon Min, Hyun-Jun Lee, Yoon Jae Lee, Sook-Hyun Lee, Kyong Sun Park, In-Hyuk Ha

**Affiliations:** a Jaseng Hospital of Korean Medicine; b Jaseng Spine and Joint Research Institute, Jaseng Medical Foundation, Gangnam-gu, Seoul, Republic of Korea.

**Keywords:** acupuncture, clinical trial protocol, low back pain, randomized controlled trial, traffic accident

## Abstract

**Background:**

Presentation of musculoskeletal symptoms, such as pain, discomfort, or disability, caused by a traffic accident (TA) is a common occurrence. However, studies on treatment and management of sudden low back pain (LBP) caused by a TA are very scarce, while studies on the effectiveness of motion style acupuncture therapy (MSAT) used on such patients are also rare. Accordingly, a randomized controlled trial (RCT) is planned to assess the effectiveness and safety of MSAT using traction (T-MSAT) for the treatment of pain and functional problems in patients with acute LBP caused by a TA.

**Methods:**

This study will be conducted at Jaseng Hospital of Korean Medicine in South Korea, using a two-armed, parallel, assessor-blinded RCT design. The study population will consist of 100 participants who will be randomly assigned in a 1:1 ratio to either the T-MSAT+integrative Korean medicine therapy (IKMT) group or IKMT control group. The treatment will be applied continuously for 3 days after admission. The primary outcome will be the difference between the numeric rating scale (NRS) scores at admission and immediately after treatment on the fourth day of admission. Secondary outcomes will include visual analogue scale (VAS) for LBP and radiating leg pain; NRS for radiating leg pain; lumbar active range of motion; Oswestry Disability Index (ODI); Patient Global Impression of Change (PGIC); the Post-traumatic Stress Disorder Checklist for DSM-5 (PCL-5-K); and 12-item short-form health survey (SF-12).

**Discussion:**

This study is a RCT to assess the effectiveness and safety of T-MSAT for acute LBP caused by a TA. The findings could be used by healthcare-related policy makers and clinicians in primary care institutions, which are frequently visited by patients suffering from LBP caused by a TA.

## 1 Introduction

Low back pain (LBP) is a common disorder, with over 80% of the world population experiencing at least 1 episode in their lifetime, and frequent recurrences may affect the quality of life of an individual.^[1]^ According to the Global Burden of Disease Study, the population affected by LBP increased significantly over a 30-year period between 1990 and 2017, as did the number of years lost due to disability (YLD) associated with LBP; thus, LBP can be viewed as a disorder that has a detrimental effect on quality of life (QoL).^[2]^ Persistent LBP restricts normal activities and causes occupational disability that not only affects the QoL of individuals, but also causes social and economic losses.^[3,4]^

The definition of acute LBP varies between studies, but typically refers to LBP that has occurred within 4 to 6 weeks, while LBP that persists for 12 weeks or more is categorized as chronic LBP.^[5]^ After the onset of acute LBP, recurrence of pain is found in more than half of cases and the overall recurrence rate is up to 75%.^[6]^ The global prevalence of LBP is 18.3% at any given time, while the 1-month and 1-year prevalence is 30.8% and 38%, respectively.^[7]^ Therefore, it is important to treat acute LBP in a timely manner to prevent progression to the chronic phase. Therapies used for LBP in the acute phase include bed rest; drug therapy, such as analgesics and herbal medicine; exercise therapy; acupuncture; electromyographic biofeedback; epidural steroid injections; and spinal manipulative therapy.^[5,6]^

With improved road conditions and advances in various modes of transportation, the number of cars owned by Koreans has increased exponentially from 530,000 in 1980 to 23.67 million in 2019. Accordingly, the number of car traffic accidents (TAs) reached 1,292,864 cases in 2019, representing an average annual increase of 3.1% since 2010, while the total number of injuries has also increased by an average of 3.3% each year.^[8]^

After a TA, various musculoskeletal injuries, internal bruises, and psychological trauma may result,^[9,10]^ while common symptoms after a TA often include neck pain or LBP.^[11,12]^ One study reported that approximately 37% of patients complained of severe LBP only 6 weeks after a TA and 23% of cases involved both neck and LBP.^[9]^ Another cohort study reported that 60.4% of patients who were treated within 30 days of an accident complained of LBP.^[13]^ Acute LBP caused by a TA could easily linger, with 1 study reporting 31% of cases presented with LBP up to 1 year after the initial injury.^[14]^ In particular, people with a history of LBP have a high risk of developing chronic LBP after an accident,^[12,15]^ and should, therefore, be managed with close attention.

Motion style acupuncture therapy (MSAT) refers to a therapeutic method in which the patient makes active or passive movements with acupuncture needles inserted in the body.^[16]^ MSAT is not only used in Korea, but also in China, for pain reduction and functional improvement for various musculoskeletal disorders, with greater efficacy than traditional acupuncture therapy.^[16–19]^ MSAT using traction (T-MSAT) utilizes a traction device to pull the body of the patient, after which, the practitioner instructs the patient to walk with the acupuncture needles already inserted to resolve lumbar musculoskeletal disorders.

Guidelines for acute LBP recommend the patient stays active and avoids bed rest, while some studies have mentioned walking or traction therapy.^[20,21]^ A study comparing T-MSAT to analgesic injection in patients with serious lumbar dysfunction reported that the pain reduction effect of MSAT was at least 5 times greater than that of the analgesic injection;^[16]^ however, studies on T-MSAT are scarce, and the evidence for the effectiveness and safety of T-MSAT is lacking.

Accordingly, this randomized controlled trial (RCT) plans to assess the effectiveness and safety of T-MSAT in patients with acute LBP caused by a TA.

## 2 Method

This study is described in accordance with the SPIRIT 2013 Statement.

### 2.1 Objectives

We plan to conduct an RCT to investigate the effectiveness and safety of T-MSAT in the improvement of conditions of patients with LBP and lumbar dysfunction.

We will investigate LBP score, lumbar dysfunction index, QoL, post-traumatic stress, and any adverse reactions, to assess the comparative effectiveness and safety between T-MSAT combination therapy and inpatient integrative Korean medicine therapy (IKMT).

### 2.2 Study design and setting

**Figure 1:**
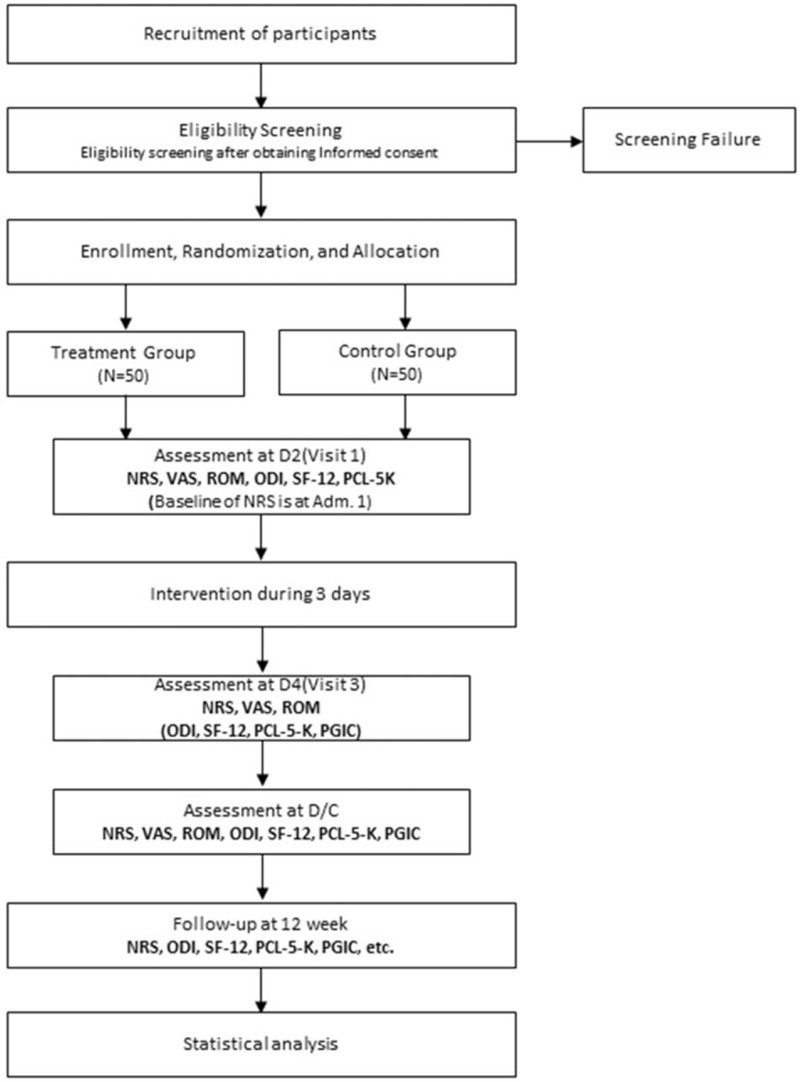
Trial flow. D/C = discharge, F/U = follow-up, LBP = lower back pain, NRS = numeric rating scale, ODI = Oswestry Disability Index, PCL-5-K = Post-traumatic Stress Disorder Checklist for DSM-5, PGIC = Patient Global Impression of Change, ROM = range of motion, SF-12 = 12-item short-form health survey, VAS = visual analogue scale.

The study trial design is detailed in Figure 1. This study will be based on a two-arm parallel, single blinded (outcome assessor blinded) RCT protocol. The clinical trial will be conducted in Jaseng Hospital of Korean Medicine (South Korea). Among people injured following TAs occurring within 1 week, 100 patients will be recruited in accordance with the inclusion and exclusion criteria (Table 1). The trial protocol was approved prior to participant recruitment by the Institutional Review Board (IRB) of the institution conducting the trial (IRB 2020-08-011). This study is registered on Clinicaltrials.gov (Clinical trials ID: NCT04554446). And the progress of the trial will be continuously updated.

**Table 1:** Inclusion and exclusion criteria.

Inclusion criteria	Exclusion criteria
1. Patients aged 19–70 years	1. Patients who have been diagnosed with a specific disease that may cause acute LBP (tumor metastasis to the spine, acute fracture, dislocation of the spinal column, etc.)
2. Patients with NRS for LBP ≥5	2. Patients with progressive neurological deficit or LBP accompanied by serious neurological symptoms
3. Patients who require inpatient treatment for acute LBP caused by a TA that occurred within one week	3. Cases in which the cause of pain is attributable to a soft tissue disorder, not the spine (tumor, fibromyalgia, rheumatoid arthritis, gout, etc.)
4. Patients who voluntarily agree to participate in the study and submit an informed consent form	4. Cases involving other chronic diseases that may interfere with the interpretation of therapeutic effects or outcomes (stroke, myocardial infarction, kidney disease, diabetic neuropathy, dementia, epilepsy, etc.)
	5. Ambulatory difficulty due to lower extremity dysfunction unrelated to LBP
	6. Patients currently taking steroids, immunosuppressants, mental illness drugs, or other drugs that may affect the study outcome
	7. Patients who are pregnant, planning to become pregnant, or breastfeeding
	8. Patients who underwent any surgery or procedure on the lumbar region within the past three weeks
	9. Patients with severe mental disorder
	10. Patients who have completed their participation in another clinical trial within the past one month or are planning to participate in another clinical trial within 12 weeks of being selected for this clinical trial or during the F/U period of this clinical trial
	11. Patients with difficulty completing the informed consent form
	12. Others who the researcher determines to be unfit to participate in the clinical trial

Among patients hospitalized at Jaseng Hospital of Korean Medicine for injuries caused by a TA, participants for this study will be recruited by posters inside the hospital, the hospital website, and physician recommendations. The recruitment will begin in September 2020.Candidates will listen to an explanation about the study given by the researcher on the first day of hospitalization (D1) and only those who voluntarily agree to participate in the study will fill out an informed consent form. Subsequently, the screening researcher will apply the inclusion and exclusion criteria (Table 1), and only 100 patients who satisfy the criteria will be selected and enrolled in the study.

The participants will be required to complete 6 visits (Vs), including the first day of hospitalization. All surveys will be conducted by face-to-face interview method, except the follow-up (F/U) survey (12 weeks after registration), which may be replaced by a telephone interview. On the second day of hospitalization (D2), the participants will be randomly assigned in a 1:1 ratio to the T-MSAT+IKMT group (N = 50) and IKMT group (N = 50) to undergo treatment. All management of participants and treatment processes will follow the trial protocol.

### 2.3 Randomization and allocation concealment

A statistics expert, not directly involved with the study, will use SAS Version 9.4 (SAS institute. Inc, Cary, NC) for block randomization to assign an equal number of participants to each group (50 per group). Randomization will be applied all 100 patients according to single-center patient recruitment/registration; the results will be sealed in envelopes and relayed to the center to be kept in a double-lock locker. The envelope will be opened in the presence of the selected participant before the treatment, after which the random allocation number will be recorded in the electronic chart. Once the allocation is made no further changes will be possible.

### 2.4 Blinding

Because the design used in this study does not allow blinding of both the patients and therapist to the respective group allocation, only the assessor will be blinded (single blind design). The assessor will not take part in the intervention. A researcher who will remain blinded to the group allocation will perform the assessment in a separate area prior to the intervention.

### 2.5 Interventions

The study population (n = 100) will be randomly allocated to 2 groups: IKMT only group (n = 50) and T-MSAT combined (T-MSAT+IKMT) therapy group (n = 50). The IKMT group will receive acupuncture, Chuna therapy, pharmacopuncture therapy, and herbal medicine; while the T-MSAT+IKMT group will also receive T-MSAT. The interventions in this study will be conducted by a physician who has been trained in advance in accordance with the reported protocol.

#### 2.5.1 Experimental group: T-MSAT+IKMT

The T-MSAT+IKMT group will take part in 3 sessions (once a day between D2 and D4). During the 3 sessions of T-MSAT, the group will also take part in IKMT sessions. A doctor of Korean medicine with over 18 months of clinical experience will use a T-MSAT device to apply traction to the patients body, after which needles will be inserted into 6 to 12 acupoints (including BL23, BL54, SP6, GB39, and Ashi acupoints), and the doctor will instruct the patient to walk. The duration of the intervention may be adjusted depending on pain or discomfort felt by the patient.

#### 2.5.2 Control group: IKMT group

The treatment for the control group will consist of only IKMT, and will include acupuncture, pharmacopuncture therapy, Chuna therapy, and herbal medicine. The treatment will be applied during hospitalization by an experienced doctor of Korean medicine.

#### 2.5.3 Co-interventions

When deemed necessary due to severe pain experienced during participation in the study (including treatment and F/U periods), use of other treatments that may affect the assessment of LBP will not be restricted. The participant will immediately notify the principal investigator who shall monitor and record adverse reactions and medication used.

### 2.6 Participants schedule

This study will be conducted as per the schedule provided in Table 2.

**Table 2:** Schedule of treatment.

	Study period					
	Screening	Active treatment				F/U
Time point	Day 1 of hospitalization	Day 2 (visit 1)	Day 3 (visit 2)	Day 4 (visit 3)	Discharge (visit 4)	12 weeks after registration (visit 5)
Enrollment						
Eligibility screening	○					
Written Informed consent	○					
Sociodemographic characteristics	○					
Information about TA^a^	○					
Medical history (post LBP, and other)	○					
Physical examination	○					
Randomized allocation		○				
Credibility and Expectancy Questionnaire		○				
Intervention						
T-MSAT+IKMT	Δ (only IKMT)	○	○	○	Δ (only IKMT)	
IKMT	○	○	○	○	○	
Assessment						
Check symptoms and Medication change		○	○	○	○	○
NRS of LBP	○	○^∗^	○	○^∗^	○	○
VAS of LBP		○^†^	○	○^†^	○	
ROM of lumbar spine		○^‡^	○	○^‡^	○	
NRS of leg pain		○^§^	○	○^§^	○	○
VAS of leg pain		○^||^	○	○^||^	○	
ODI		○		○^¶^	○	○
SF-12		○		○^¶^	○	○
PGIC				○^¶^	○	○
PCL-5-K		○		○^¶^	○	○
Diagnostic imaging^b^						○
Details of F/U^c^						○
Adverse events		○	○	○	○	○

### 2.7 Outcome measures

The outcomes will be measured at each time point during 12 weeks of the study period, and the primary endpoint is D4 (V3) when the treatment is completed.

#### 2.7.1 Primary outcome

The primary outcome will be the difference between numeric rating scale (NRS) for LBP at admission and at completion of treatment on D4. NRS is a pain scale used to express the subjective pain felt by the patient as an objective numerical value. The patient is instructed to select a number between 0 (no pain) and 10 (worst pain imaginable) that best describes their current pain level.^[22,23]^

#### 2.7.2 Secondary outcomes

Secondary outcomes will be assessed by NRS for radiating leg pain; visual analogue scale (VAS) for lumbar and radiating leg pain; lumbar active range of motion (aROM); Oswestry Disability Index (ODI); 12-item short-form health survey (SF-12); Post-traumatic Stress Disorder Checklist for DSM-5 (PCL-5-K); and Patient Global Impression of Change (PGIC). In addition, F/U investigation will be conducted at 12 weeks after study registration to assess: NRS for leg and LBP; NRS; ODI; SF-12; PCL-5-K; and PGIC, and radiological findings from lumbar imaging performed after TA (L-spine X-ray, L-spine MRI, and L-spine CT) will be recorded, while accident settlement, completion of treatment, and return to work will also be investigated.

VAS for LBP and radiating leg pain: VAS is an assessment tool for recording pain felt by a patient using a line that is 100 mm long, with one end indicating no pain and the other end indicating worst pain imaginable.^[22,24]^ To identify the level of LBP and radiating leg pain felt, the patient will be instructed to indicate a point on the line that best describes his or her level of pain.

NRS of leg pain: The patient will be instructed to select a numerical value between 0 (no pain) and 10 (worst pain imaginable) that best describes the subjective radiating leg pain he or she is feeling.

Lumbar aROM by physical examination: The lumbar aROM that allows the patient to move without pain will be measured. The lumbar aROM in 6 directions (flexion, extension, right lateral flexion, left lateral flexion, right rotation, and left rotation) will be measured.

ODI: The low back functional disability of patient will be assessed using the ODI questionnaire.^[25]^ ODI is a 10-item tool developed for assessing level of low back functional disability, with each item divided into 6 stages and assigned 0 to 5 points. Higher total scores indicate more severe disability and this study will use the official Korean version of the ODI questionnaire.^[26]^

Korean version of health-related QoL (HRQoL) assessment scale (SF-12 v2): SF-12 v2 is a questionnaire used to assess HRQoL, comprising 12 items in 8 categories (physical function, role-physical, bodily pain, general health, vitality, social function, role-emotional, and mental health). This tool will used in this study to assess the level of functional health and life, with higher scores indicating better HRQoL. For the Korean version of SF-12, Kim et al tested the reliability and validity to measure HRQoL in 1000 Koreans.^[27]^

PCL-5-K: Post-traumatic stress disorder (PTSD) checklist (PCL) is one of the most widely used self-reporting scale for assessing PTSD. This tool will be used in this study to measure the degree of stress in the patient caused by a traumatic factor in the form of a TA. The study will use the Korean version of the checklist adapted into Korean by Kim et al^[28]^ and subsequently tested for reliability and validity.

Global assessment scale (PGIC): PGIC is a method used to subjectively assess the degree of improvement in a patients condition, divided into 7 steps^[29,30]^: 1 = very much improved; 2 = much improved; 3 = minimally improved; 4 = no change; 5 = minimally worse; 6 = much worse; and 7 = very much worse. In this study, PGIC will be measured from the start of study to discharge, and during the F/U after 12 weeks.

#### 2.7.3 Treatment credibility and expectancy scale

To assess the expectation that the participants have about the treatment, a 9-point Likert scale is used for assessment. Prior to the first treatment, the participants will be asked “how much do you expect this treatment to alleviate your symptoms?” The response to this question will be scored (1 = not at all; 5 = somewhat; and 9 = very much).

#### 2.7.4 Medication use

The type, dose, and frequency of medication used by the participants during the study period will be investigated during the visits.

#### 2.7.5 Adverse events

An adverse event (AE) refers to any undesirable and unintended sign, symptom, or disorder that appears during the clinical trial. AEs will be checked during each visit and recorded without exception. AEs do not necessarily need to show a causality with the treatment applied in this study. The researcher will analyze the frequency of AEs and serious AEs (SAEs) suspected to be associated with the treatment. Collected safety data will be adequately summarized and all SAEs will be described in detail. An SAE^[31]^ is an AE that:

1. causes death or is life-threatening;
2. requires hospitalization or prolongs hospitalization;
3. causes persistent or significant disability or dysfunction; or
4. an otherwise medically important situation.

The researcher will assess the causality between AEs that occur and each treatment method by a 6-step scale in accordance with the WHO-UMC causality assessment system^[32]^: 1 = definitely related; 2 = probably related; 3 = possibly related; 4 = probably not related; 5 = definitely not related; and 6 = unknown. The severity of AEs will be classified into 3 levels according to the Spilker classification^[33]^:

1. mild = an AE that does not significantly impair daily activities (function) nor require additional medical intervention;
2. moderate = an AE that significantly impairs the normal life (function) of the participant, and may require additional medical intervention but resolves afterwards; and
3. severe = an SAE that requires intense medical intervention, and results in sequelae.

Actions for procedure after an AE will be classified and recorded as follows:

1. not applicable (no effect on the procedure);
2. temporary discontinuation; and
3. permanent discontinuation.

The F/U outcome of the AE will be classified and recorded as follows:

1. recovered, no sequelae;
2. recovered with sequelae;
3. recovering, persistent AE, no progress;
4. no recovery, presence of AE, progressing;
5. death; and
6. unknown.

Actions taken other than planned treatment following an AE will be classified and recorded as follows:

1. none;
2. drug therapy;
3. hospitalization/prolonged hospitalization;
4. therapeutic or diagnostic procedure; and
5. others.

To prevent random group allocation being revealed during AE assessment, AE assessments will be reported in a separate case report by a designated investigator. The severity and causality of AEs and actions taken will be assessed according to the procedure described above. In this study, safety of the treatment will be assessed by identifying the frequency and severity of AEs and comparing them between the 2 groups.

### 2.8 Sample size

The null hypothesis of this study is that applying additional T-MSAT to patients with acute LBP will not show any difference in pain between the experimental and control groups. To test this hypothesis, ANCOVA will be used as the primary analysis.

The significance level was set to α=.05 (two-sided test); type 2 error (β) was set to .2; and statistical power was set to 80%. The effect size calculated in a previous study^[16]^ for determining the sample size was .58. When the sample size was calculated based on these parameters, 48 was determined to be the sample size required for each group. Considering that this study will use ANCOVA as the main analysis and the correlation will be set to .5, at least 37 participants would be needed per group.^[34]^ Assuming a drop-out rate of 25%, the goal will be to recruit and enroll a total of 100 participants.

### 2.9 Patient and public involvement statement

Neither patients nor the public were involved in the development of the research questions, selection of outcome measures, study design, or study conduct.

### 2.10 Data management & analysis plan

#### 2.10.1 Data collection and management

This study will use paper case reports and an in-house standard operating procedure (SOP) will be prepared for training the researchers, including assessors and physicians, about case report writing, data input, and treatment procedures. The researchers will enter the data in duplicate. Only the investigator in charge of data management will have access to all the data.

#### 2.10.2 Statistical analysis

This study is an RCT on patients with acute LBP caused by a TA, in which we will conduct between-group and within-group comparative analyses on effectiveness and safety indicators obtained from the participants. This study will conduct both intention-to-treat (ITT) and per-protocol (PP) analyses. ITT analysis will be used as the primary analysis, while PP analyses will be performed separately on participants who received 3 or more treatments. Missing values will be processed by multiple imputation as the main method, with the statistics expert blinded. Moreover, last observation carried forward (LOCF) will be used for sensitivity analysis.

The demographic characteristics of the participants and treatment expectancy will be assessed for each group. Continuous variables will be expressed as mean (standard deviation) or median (quartile) and the difference between the 2 groups will be compared by student *t* test. Moreover, categorical variables will be expressed as frequency (%) and tested by Chi-Squared test or Fishers exact test.

The endpoints of this study could be viewed as the amount of change in continuous outcomes (NRS, VAS, ODI, SF-12, aROM, and PCL-5-K) between the baseline and each time point. ANCOVA will be performed as the main analysis, which will use the pre-treatment (baseline) values of each variable and covariant factors selected by group at baseline as covariates, and group as a fixed factor. For additional analysis, a linear mixed model will be used to test the differences in trend changes at each visit. To compare the total amount of each outcome value within the study period for the 2 groups, the areas under the curve for each time point after random allocation will be calculated and compared by student *t* test. In addition, the percentage of patients at time points when NRS and VAS (LBP indicators) decrease by 50% below the baseline will be compared and analyzed. Kaplan–Meier survival analysis will be performed to compare the time required to achieve recovery from LBP, as indicated by pain indicators decreasing by 50% and the curves will be compared by log-rank test. Additionally, Cox proportional hazard model will be used to compare hazard ratios. We also plan to perform subgroup analyses for exploratory purposes, to compare the differences in the level of pain improvement in the T-MSAT and control groups. The significance level for all analyses will be set to .05. SAS 9.4 (© SAS Institute, Inc, Cary, NC, USA) or R studio 1.1.463 (© 2009–2018 RStudio, Inc,Boston, MA, USA) will be used for all statistical analysis and significance will be determined based on *P* < .05.

### 2.11 Data monitoring and safety monitoring

To assure the safety of participants and the integrity of the study data, the monitor will compare the case records with supporting documents and review the participant safety data during monitoring. Monitoring will take place as: initial monitoring at the time of participant screening; one or more intermediate checks during the trial; and final monitoring upon completion of the clinical trial. The number of monitoring sessions may be increased or decreased based on discussions with the researcher.

### 2.12 Stopping rules

The status of whether the clinical trial was completed by each participant will be recorded, and for cases involving discontinuation of the intervention or observations prior to completion, the time point of discontinuation and the reason(s) will be recorded in the case report. The participants may voluntarily withdraw from the clinical trial at any time and the researcher may dismiss a participant at his or her discretion. Discontinuation during the clinical trial may occur due to the following reasons: discovery of a disease that may affect the determination of the outcomes of the study; a request by the participant to withdraw; confirmed pregnancy while under treatment; identification of any problem in applying the intervention for LBP to any participant; and other reasons the researchers determine to be unsuitable to continue the study. When the intervention is discontinued, follow-up observations may continue as agreed upon by the patient. Participants who drop out early due to AE may receive appropriate treatment for such AE when necessary, and for early drop out due to AE that has a causal relationship with this study, the researcher may continue to assess the participant until the AE is resolved or determined to be permanent.

### 2.13 Ancillary and post-trial care

In the case of direct harm in relation with this study, appropriate medical care may be obtained as determined by the investigator. Compensations for any damage would be proceeded according to the pre-designated insurance policy related to the study. All participants will be provided with an emergency contact number to reach study investigators so that they can receive the necessary support when they have any question or problem.

## 3 Discussion

MSAT is a therapeutic technique used for alleviation of acute pain and recovery of functional state associated with musculoskeletal disorders, by applying acupuncture to the area being treated and having the patient generate active or passive movements. Studies of MSAT include: a RCT that reports improvements in cervical pain and dysfunction through MSAT in patients who suffered whiplash injury after a TA^[17]^; a multi-center RCT that demonstrated that using T-MSAT on patients with acute LBP and severe dysfunction produced a therapeutic effect that was more than 5 times greater than that of conventional analgesic injection^[16]^; and RCTs that showed greater pain reduction and ROM improvement from using MSAT on patients with acute LBP, as compared to loxoprofen or physical therapy.^[35,36]^ T-MSAT used in this study is a type of MSAT that uses traction, and while it is similar to MSAT used in the study by Shin et al.^[16]^, it uses a specially-made device, instead of a person, to pull the patients body; and has the patient repeat active walking under the supervision of a physician after applying acupuncture to acupoints corresponding to LBP, to achieve reduction in LBP and improvement in lumbar dysfunction. With regard to T-MSAT, there is a study that applied T-MSAT and tested its effect on inpatients with lumbar dysfunction,^[37]^ but that study had several limitations, including retrospectively analyzing medical records and not clearly classifying LBP.

Studies on MSAT still lack high-level evidence, and studies on T-MSAT are almost non-existent. Therefore, our study could be viewed as the first RCT to identify the effectiveness and safety of T-MSAT applied to acute LBP following an accident.

For recruitment of participants for the study, drop-out rate and sample size were calculated based on previous RCTs that used MSAT to treat acute LBP. In contrast to existing studies that did not apply criteria for differentiating acute and chronic cases,^[37]^ or others that established the cause based on comprehensive time of onset,^[16]^ this study attempted to recruit participants based on a clear criterion of acute LBP occurring within one week after a TA. Consequently, a comparison to other studies on acute LBP would be easy since the patient population was clearly established based on involvement of trauma.

For selection of the control group, socioeconomic choices of TA patients who are admitted to Korean medicine hospitals were considered, and the limitations of treatments patients can receive depending on insurance coverage could present an ethical problem. Accordingly, only the IKMT group was designated as the control group to include all patients who received acupuncture, Chuna therapy, pharmacopuncture, and herbal medicine.

The study period is set to 3 months, including the F/U period, during which time clinical progress related to LBP will be observed, and car insurance settlements will be checked. The study may also investigate whether the car insurance settlement is affected by any injuries discovered through radiological imaging after the accident.

However, this study has some limitations. Firstly, the study design does not allow the person applying the treatment to be blinded. To address this, the assessor will be different to the treating physician, and blinded. Secondly, because a history of LBP is known to be a key predictor of LBP after an accident,^[15]^ it is important to minimize the involvement of any bias associated with this. Therefore, this study will randomly assign the registered participants to the treatment groups, and a history of LBP prior to the accident will be investigated separately. Thirdly, the study population will include only patients who were hospitalized within one week after an accident; thus, it would be difficult to generalize the findings for outpatients who were not hospitalized, or patients with acute LBP more than 7 days after an accident. Nonetheless, this study will assist in the planning of future studies pertaining to the assessment of the effectiveness of T-MSAT for other disorders.

The effectiveness and safety of T-MSAT, that have not been studied to date, will be identified through this clinical trial and the findings could be used to develop a treatment method that prevents acute LBP after an accident from progressing to chronic pain. This will assist in expanding the treatment options for acute LBP. We hope that the findings may be useful for clinicians working in healthcare institutions and medical insurance-related policy experts.

## Author contributions

**Conceptualization:** Jeong-Hun Han, Byung-Hak Park.

**Investigation:** Jin-Hun Park, Tae-Woon Min, Hyun-Jun Lee.

**Methodology:** Yoon Jae Lee, Sook-Hyun Lee, Kyoung Sun Park.

**Project administration:** In-Hyuk Ha.

**Supervision:** Yoon Jae Lee, In-Hyuk Ha.

**Writing – original draft:** Jeong-Hun Han, Byung-Hak Park.

**Writing – review & editing:** Jeong-Hun Han, Byung-Hak Park.
